# Autochthonous Probiotics in Meat Products: Selection, Identification, and Their Use as Starter Culture

**DOI:** 10.3390/microorganisms8111833

**Published:** 2020-11-21

**Authors:** Paulo E. S. Munekata, Mirian Pateiro, Wangang Zhang, Rubén Domínguez, Lujuan Xing, Elena Movilla Fierro, José M. Lorenzo

**Affiliations:** 1 Centro Tecnológico de la Carne de Galicia, rúa Galicia n° 4, Parque Tecnológico de Galicia, San Cibrao das Viñas, 32900 Ourense, Spain; paulosichetti@ceteca.net (P.E.S.M.); mirianpateiro@ceteca.net (M.P.); rubendominguez@ceteca.net (R.D.); 2 Key Laboratory of Meat Processing and Quality Control, Ministry of Education China, Jiangsu Collaborative Innovation Center of Meat Production and Processing, Quality and Safety Control, College of Food Science and Technology, Nanjing Agricultural University, Nanjing 210095, China; wangang.zhang@njau.edu.cn (W.Z.); lujuanxing@njau.edu.cn (L.X.); 3 Complejo Hospitalario Universitario de Ourense, 32005 Ourense, Spain; elemofierro@hotmail.com; 4 Área de Tecnología de los Alimentos, Facultad de Ciencias de Ourense, Universidad de Vigo, 32004 Ourense, Spain

**Keywords:** functional meat products, LAB, autochthonous bacteria, inoculum, quality

## Abstract

The increasing demand for functional food is pushing the food industry to innovate the conventional and well-known foods. Producing functional foods, especially with probiotics in meat products, is an intricate and multistage task that involves: the selection of microorganisms with probiotic potential, the identification at strain level, and the evaluation of probiotic strains in the processing of meat products. The resistance to digestion, followed by the successful colonization in the small intestine and the safety are the main criteria used to select and identify (at strain level) a probiotic, as reported in recent studies about the autochthonous microbiota of meat products. Further insertion (as starter culture) in a meat system for fermentation is the simplest approach to obtain a probiotic meat product. Among the innumerous microorganisms naturally found in meat products, lactic acid bacteria (LAB) play a central role by fitting in both probiotic and meat products processing criteria.

## 1. Introduction

The fermentation of meat dates back to centuries ago when meat cuts were spontaneously fermented by its natural (autochthonous) microbiota without any control of processing conditions to preserve meat [1]. In European countries, this practice is believed to have originated in the Mediterranean countries and posteriorly spread to other countries where particular aspects of each location influenced the characteristics of each product, which include the environmental microbiota, ingredients, and practices in the processing [2]. Although the characteristics of traditionally produced (texture, color, and flavor, for instance) are highly appreciated, producing fermented meat products using autochthonous microorganisms (naturally present in meat or from environment where the processing is carried out) is an important issue in terms of public health [3,4]. However, consumers perceive traditionally produced meat products with higher quality even though the risk associated with their consumption (growth of pathogenic bacteria and accumulation of toxins and harmful compounds) [4].

Improving the characteristics of meat products is another important aspect that modern consumers consider relevant in the moment of purchase, especially when a health benefit is the additional attribute [5]. Moreover, the combination of traditional practices with modern strategies to produce meat products should not be seen as contrasting concepts [6]. However, the increasing interest in functional food products has brought important challenges for the food industry, especially in muscle product category [7,8]. The use of probiotics is part of the successful strategies to improve foods from their conventional or traditional production form to functional food category, especially for fermented food products [9]. The ingestion of living microorganisms (also known as probiotics) in adequate amount (6–7 log CFU/g) has been associated with several health benefits for the host. Functional foods can be defined as food products with additional benefits beyond those related to basic nutrients [10,11].

In modern times, the control of the processing conditions and quality of fermented meat products has greatly improved, especially because of the use of specific microorganisms such as the lactic acid bacteria (LAB) [12,13]. LAB plays a central role as one of the most studied groups of microorganisms for the development of functional foods because of their benefits to human health, potential to prevent the formation of toxic compounds (such as biogenic amines), and also being Generally Recognized as Safe (GRAS) [14,15,16]. Moreover, LAB has been associated with health benefits such as improving immunity, anti-oxidative capacity, and peristalsis in healthy subjects [17], improving the glycemic control and indicators of cardiovascular diseases in diabetic nephropathy patients [18], and reducing intestinal inflammation in ulcerative colitis patients [19].

**Figure 1:**
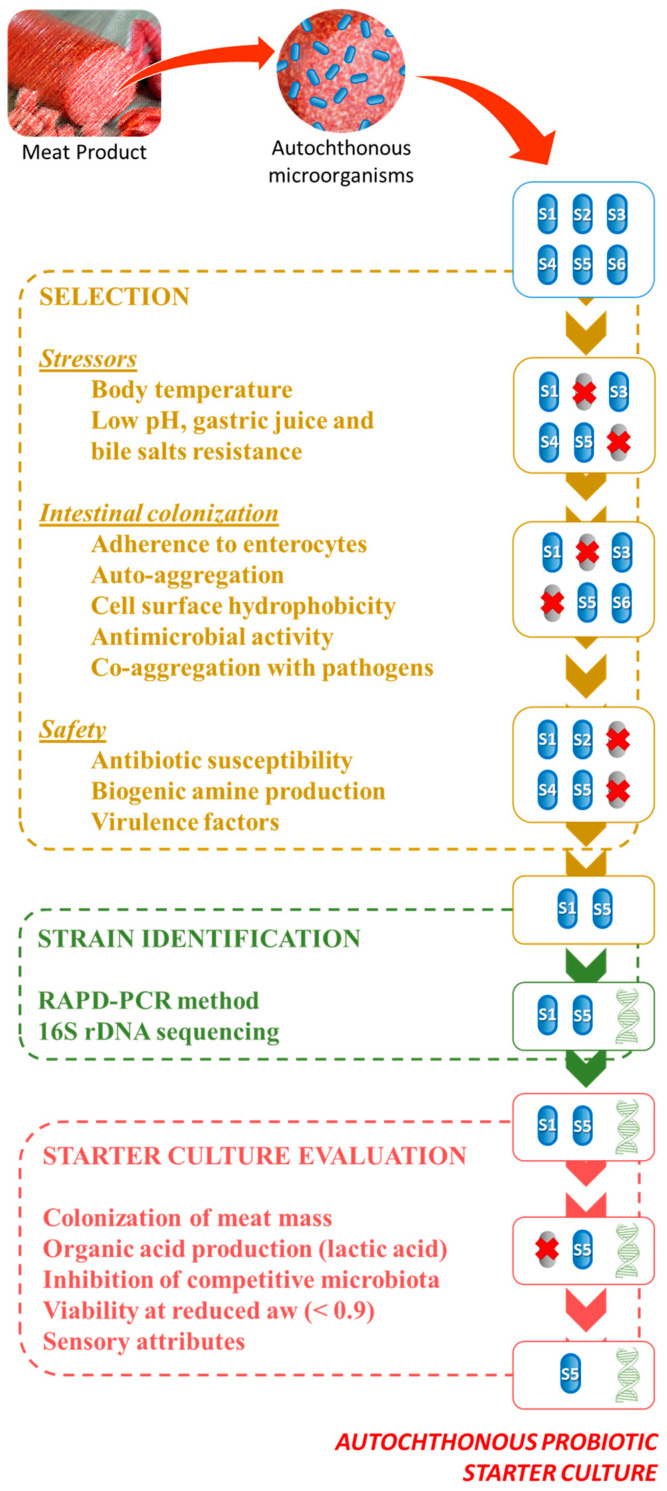
Schematic representation of the selection, identification, and evaluation of starter culture potential of meat products autochthonous microorganisms.

The use of starter cultures can bring several benefits in comparison to spontaneous fermentation: better control of the fermentation as a whole, reduce the ripening time, reduce the possibility of pathogenic microorganism growth, and also improve the preservation of quality between batches [20,21]. However, selecting an adequate starter culture for the development of a functional meat product is a challenging task because of the complexity of each step and the numerous assays required. The Figure 1 indicates a schematic representation of the screening approach for the selection of probiotic starter cultures from autochthonous microbiota of meat products.

The first step consists in the evaluation of the probiotic potential. In this stage, the influence of stressors of the digestion, intestinal colonization, and safety aspects is decisive to define the probiotic viability of an isolate [11]. Once the dietary probiotics are ingested, the microorganisms are exposed to a hostile environment including the body temperature, gastric juice, hydrochloric acid, and bile salts. Prevailing to these stressors is an important indicator that viable cells can reach the intestine. In the following criteria stage, the viable cells are expected to colonize the intestine. This task is achieved by adhering to epithelial cells, auto-aggregation (small agglomerations of microbial cells), having high cell surface hydrophobicity, inhibiting the growth of pathogenic microorganisms that compete for the limited resources in the intestine, and also by co-aggregating to pathogenic cell and facilitating its release in the feces, for instance [11,22].

Another relevant aspect related to the screening for probiotic strains in autochthonous populations is the identification of species and the strains of potential candidates. The characterization at species level does not provide sufficient information to distinguish probiotics [23]. In this sense, the characterization of a potential probiotic isolate can be carried out by either nucleic acid or throughout cell activity assessment. The polymerase chain reaction (PCR)-based and 16S rRNA methods can be cited as relevant methods for the evaluation of nucleic acids of probiotic microorganisms [24].

Once the screening of isolates is complete and the probiotic strains are defined, the evaluation of these strains from the food technology point of view is necessary. In other words, the selected probiotic strains are used in the simplest strategy: starter cultures. The strategic selection of starter cultures consists on evaluating indicators: the fast and persistent colonization of meat mass, the production of organic acids (especially lactic acid), the inhibition of competitive microbiota (both spoilage and pathogenic microorganisms), prevailing at a reduced water activity (aw), and also preserving or enhancing the sensory attributes of fermented meat product [25]. Taking into account the relevant and increasing information published recently about the role of autochthonous probiotic microorganism found in meat products, this review aims to discuss the selection, identification, and evaluation as starter culture of meat products autochthonous microorganisms.

## 2. Characterization of Autochthonous Probiotics Found in Meat Products

### 2.1. Selection Criteria for Probiotic Strains

The selection of probiotic microorganisms takes into account the stressor and the expected effect during digestion and colonization of gut. The criteria to select probiotic microorganisms comprises multiple aspects that include the influence of digestion on viability, metabolism, and growth; the adherence to enterocytes; the capacity to inhibit competitive microbiota (especially pathogenic bacteria); the low harmful potential (virulence factors and biosynthesis of biogenic amines, for instance); and the susceptibility to antibiotics of isolated strains [11,23].

Regarding the potential probiotic microorganism naturally found in meat products, recent studies characterize promising candidates (Table 1). The capacity to prevail under unfavorable conditions such as those imposed by digestion is the first criterion to select probiotic strains. For instance, the evaluation 42 LAB isolated from *Ciauscolo* salami (traditional Italian fermented sausage) indicated most strains were capable to survive to low pH and bile salts [26]. The authors indicated that *Pediococcus pentosaceus* 62781-3, 46035-1, 46035-4 and *Leuconostoc mesenteroides* 14324-8 strains were capable to resist hydrochloric acid (pH 2.5) or bile salts (pH 7.2) for 3 h in MRS broth.

In a related experiment with Harbin dry sausages (traditional Chinese fermented sausage), four isolates (*P. pentosaceus* R1, *Lactobacillus brevis* R4, *Lactobacillus curvatus* R5, and *Lactobacillus fermentum* R6) were evaluated regarding the capacity to survive simulated gastric digestion in stomach (pH 3.0) and gut (pH 8.0) [27]. The isolates *L. brevis* R4 and *L. fermentum* R6 displayed the highest survival rates for digestion in both stomach and gut. Likewise, two strains of *Lactobacillus plantarum* (CB9 and CB10) were selected from the natural microbial community of cured beef because of their capacity to survive after being exposed to pH 2.0 and bile salts [28]. A similar outcome was reported for *Enterococcus faecium* 120 that displayed both acidic and bile salt resistance in simulated gastric juice [29].

A recent study evaluated the acidic and bile resistance of five LAB isolates (*Lactococcus lactis* subsp. *cremoris* CTCa 204, *L. lactis* subsp. *hordiniae* CTC 483, *L. lactis* subsp. *cremoris* CTC 484, *L. plantarum* CTC 368, and *L. plantarum* CTC 469) obtained from different meat products [30]. Although all strains were resistant to an acidic environment (on pH 1–5 for up 3 h), the same effect was not observed for bile salt test wherein all strains were sensible to both bile salt concentration (from 0.1 to 2.0%) and exposure time (up to 3 h). Similarly, the study carried out by Petrović et al. [31] evaluated 21 *E. faecium* isolates regarding the capacity to prevail in low-pH medium and with bile salts in simulated gastric juice. According to authors, all the strains presented acid tolerance but only two strains (sk7-5 and sk9-15) were resistant to bile salts. In a recent study, the resistance of *Pediococcus acidilactici* CE51 (isolated from a meat sausage) to low pH and bile salts was evaluated [32]. The authors indicated that this isolate was resistant to acid (pH 2.0, 2.5, and 3.5) but it was affected by bile salts (0.9%). A related experiment indicated that *Staphylococcus* sp. DBOCP6 (isolated from fermented meat products) displayed capacity to resist the stress induced by gastrointestinal digestion [33].

The study carried out by Klayraung et al. [34] observed that 36 and 66 strains (from a total of 169 isolates from different *Lactobacillus* spp.) were resistant to acid and bile salts, respectively. A related experiment with isolates from pork sausages indicated that most of 32 *Lactobacillus* spp. strains displayed potential to survive the digestion and reach the intestine (survival rate > 90%) [35]. In the same line of research, the experiment carried out by Topçu, Kaya, and Kaban [36] with isolates from traditionally produced Pastırma indicated that all *P. pentosaceus* strains (K7, K41, K44, K51, and K81) and *P. acidilactici* K99 were capable to resist simulated gastric and intestinal fluid.

Yuksekdag and Aslim [37] investigated the natural microbial community in Sucuk for potential probiotics. Among the selected isolates, *P. pentosaceus* Z12P and Z13P strains displayed the highest capacity to resist the impact of low pH and bile salts. In a related experiment, Zommiti et al. [38] carried out a similar experiment with *E. faecium* strains isolated from dried Ossban (a Tunisian fermented meat product). The five strains selected for probiotic evaluation displayed good potential to resist the stress imposed by low pH and bile. Scandinavian-type fermented sausages are also relevant sources of autochthonous probiotic as indicated by Klingberg et al. [39]. According to the authors, most of the isolated microorganisms (*Lactobacillus* spp. and *P. pentosaceus*) displayed potential to grow in acidic environment and in the presence of bile salts.

**Table 1:** Autochthonous probiotic strains found in meat products.

Source of Probiotic Microorganisms	Probiotic Selection Assays	Isolated Microorganisms	Potential Probiotics	Ref.
*Ciauscolo* salami (traditional Italian fermented sausage)	Resistance to low pH and bile salts, cell adhesion, and antibiotic resistance	42 LAB ^1^ isolates comprising: *Carnobacterium spp.*, *Enterococcus faecalis*, *Lactobacillus brevis*, *Lactobacillus casei*, *Lactobacillus johnsonii*, *Lactococcus lactis*, *Lactobacillus paracasei*, *Lactobacillus paraplantarum*, *Lactobacillus sakei*, *Lactococcus* spp, *Leuconostoc mesenteroides*, *Pediococcus pentosaceus*, and *Weissella hellenica* strains	*P. pentosaceus* 62781-3, 46035-1, and 46035-4, and *L. mesenteroides* 14324-8	[26]
Traditional Portuguese fermented meat products	Resistance to low pH, bile salts, and body temperature; antimicrobial activity; and biogenic amine production	*Enterococcus faecium* 85, 101, 119, and 120	*E. faecium* 120	[29]
Harbin dry sausages (traditional Chinese fermented sausage)	Resistance to gastric transit and bile salts, auto-aggregation, cell adhesion, and hydrophobicity	*P. pentosaceus* R1, *Lactobacillus brevis* R4, *Lactobacillus curvatus* R5 and *Lactobacillus fermentum* R6	*L. brevis* R4	[27]
Meat products	Resistance to low pH, bile salts, and body temperature; biofilm formation; virulence factors; antibiotic resistance; and biogenic amine production	*L. lactis* subsp. *cremoris* CTCa 204, *L. lactis* subsp. *hordiniae* CTC 483, *L. lactis* subsp. *cremoris* CTC 484, *Lactobacillus plantarum* CTC 368, and *L. plantarum* CTC 469	*L. lactis* CTC 204 and *L. plantarum* CTC 368 strains	[30]
Cured beef	Resistance to low pH and bile salts; antimicrobial activity; auto- and co-aggregation; cell adhesion and hydrophobicity; hemolytic activity; and antibiotic resistance	*L. plantarum* (CB9 and CB10) and *Weissella cibaria* CB12	*L. plantarum* CB9 and CB10 strains	[28]
*Sokobanja* sausage (traditional Serbian sausage)	Resistance to simulated gastrointestinal digestion; antimicrobial activity; biogenic amine production; and antibiotic resistance	*E. faecium* sk6-1 and -17; sk7-5, 7 and 8; sk8-1, 2, 4, 5, 7, 12, 13, 17 and 20; sk9-3, 11 and 15; sk10-1, 7, 10 and 12	*E. faecium* sk7-5, sk7-8 and sk9-15	[31]
Meat sausage	Resistance to low pH and bile salts; antimicrobial activity; and antibiotic resistance	*Pediococcus acidilactici* CE51	Suitable probiotic characteristics	[32]
Fermented pork sausages	Resistance to simulated gastrointestinal digestion, cell hydrophobicity, antimicrobial activity, and antibiotic resistance	169 *Lactobacillus* spp. strains (*L. curvatus, Lactobacillus reuteri, L. plantarum, Lactobacillus parapentarum, L. pentosus, Lactobacillus keferi, L. fermentum, Lactobacillus animalis, Lactobacillus mucosae, Lactobacillus aviaries* ssp. *aviaries, L. salivarius* ssp. *salicinus, L. salivarius* ssp. *salivarius, Lactobacillus hilgardii*, and *Lactobacillus panis*)	*L. fermentum* 3007 and 3010 strains	[34]
Pork sausages	Resistance to simulated gastrointestinal digestion, cell hydrophobicity, auto- and co-aggregation, hemolytic activity, biogenic amine production, and antibiotic resistance	32 *Lactobacillus* spp. strains	*L. plantarum* UFLA SAU 14, 20, 34, 52, 91, 172, 185, 187, 238, and 258	[35]
Pastırma (Turkish cured beef product)	Resistance to simulated gastrointestinal digestion, cell hydrophobicity, auto- and co-aggregation, and cell adhesion	*P. pentosaceus* K7, K41, K44, K51, and K81 and *P. acidilactici* K99	*P. pentosaceus* K41 and K44 and *P. acidilactici* K99	[36]
Sucuk (Turkish fermented sausage)	Resistance to simulated gastrointestinal digestion, antimicrobial activity, auto- and co-aggregation, and antibiotic resistance	*P. pentosaceus* Z9P, Z12P, and Z13P, *P. acidilactici* Z10P, and *P. dextrinicus* Z11P	*P. pentosaceus* Z12P and Z13P	[37]
Dried Ossban (Tunisian fermented meat product)	Resistance to simulated gastrointestinal digestion, auto-aggregation, cell adhesion, virulence factors, biogenic amine production, bacteriocin production, antimicrobial activity, and antibiotic resistance	*E. faecium* strains MZF1, MZF2, MZF3, MZF4, and MZF5	All strains	[38]
Scandinavian-type fermented sausages	Resistance to simulated gastrointestinal digestion, cell adhesion, and antimicrobial activity	*Lactobacillus alimentarius MF1297, Lactobacillus farciminis DC11 and MF1288, Lactobacillus pentosus MF1300, L. plantarum DC13, MF1291, MF1298, Lactobacillus rhamnosus DC8, L. sakei MF1295, MF1296, Lactobacillus salivarius DC2, DC4, DC5,* and *P. pentosaceus* *DC12*	*L. pentosus* MF1300 and *L. plantarum MF1291* and *MF1298* strains	[39]
Spanish dry-cured sausages	Resistance to simulated intestinal digestion, biofilm formation; virulence factors, biogenic amine production, and antibiotic resistance	46 LAB strains (*E. faecium*, *Lactobacillus coryniformis*, *L. paracasei*, *L. plantarum*, and *L. sakei*)	*L. paracasei* Al-128 and *L. sakei* Al-143	[40]
Slavonski kulen sausage (traditional Croatian sausage)	Resistance to simulated intestinal digestion, antimicrobial activity, enterotoxin production, and antibiotic resistance	*L. plantarum* 1 K, *L. delbrueckii* 2 K, *L. mesenteroides* 6K1, *L. acidophilus* 7K2, *S. xylosus* 4K1, *S. warneri* 3K1, *S. lentus* 6K2, and *S. auricularis* 7K1	All LAB and *S. xylosus* 4K1, *S. warneri* 3K1 strains	[41]
Slovak traditional sausages	Resistance to simulated intestinal digestion, antimicrobial activity, bacteriocin production, cell adhesion, biogenic amine production, and antibiotic resistance	*S. xylosus* and *S. carnosus* strains	*S. xylosus* SO3/1M/1/2	[42]
Indian fermented meat	Resistance to simulated gastrointestinal digestion, cell hydrophobicity, auto-aggregation, antimicrobial activity, hemolytic activity, and antibiotic resistance	*Staphylococcus* sp. DBOCP6	Suitable probiotic characteristics	[33]
Fermented meat products	Resistance to simulated gastrointestinal digestion, hemolytic activity, cell adhesion, cholesterol-lowering property, and antibiotic resistance	12 γ-aminobutyric acid-producing strains	*P. pentosaceus* HN8 and *L. namurensis* NH2	[43]
Vienna sausages	Resistance to simulated gastrointestinal digestion, cell hydrophobicity, cell adhesion, auto- and co-aggregation, and antibiotic resistance	*E. faecium* UAM1, UAM2, UAM3, UAM4, UAM5, and UAM6	*E. faecium* UAM1	[44]
Iberian dry fermented sausages	Resistance to simulated gastrointestinal digestion	15 LAB and bifidobacteria strains (*Lactobacillus* spp., *Bifidobacteria* spp., *Lactococcus* spp., and *Enterococcus* spp.)	*P. acidilactici* KKA and UGA146-3 and *E. faecium* CICC 6078, CK1013, and IDCC 2102	[45]
Traditional dry fermented sausages	Resistance to simulated gastrointestinal digestion	20 *Lactobacillus* spp. strains	*L. brevis* AY318799, AY318801, and AY318804, *L. curvatus* AY318826, *L. fermentum* AY318825, *L. paracasei* ssp. *paracasei* AY318806, AY318809, and AY318824, and *L. plantarum* AY318822	[46]

^1^ LAB: lactic acid bacteria.

Landeta et al. [40] reported that isolated *L. sakei* strains were more resistant to bile salts than *E. faecium* strains (all obtained from Spanish dry-cured sausages). Moreover, the authors also observed that *L. plantarum* AI-122 and AI-148 strains were the most resistant among all isolates. In a similar way, Babić et al. [41] investigated the microbiota of Slavonski kulen sausage for potential probiotics. According to the authors, the four isolated LAB (*L. plantarum* 1 K, *L. delbrueckii* 2 K, *L. mesenteroides* 6K1, and *L. acidophilus* 7K2) and two strains of *Staphylococcus* (*S. xylosus* 4K1 and *S. warneri* 3K1) displayed capacity to resist the action of bile salts.

Isolates (*S. xylosus* and *S.carnosus*) with capacity to resist the stress imposed by bile salts were also reported in Slovak traditional sausages [42]. The authors of this study indicated that the ability to survive varied between 54 and 99%. Likewise, Ruiz-Moyano et al. [45] observed that among the 15 isolated microorganisms from Iberian dry fermented sausages *P. acidilactici* KKA and UGA146-3 and *E. faecium* CICC 6078, CK1013, and IDCC 2102 displayed potential to grow after a simulated gastrointestinal digestion.

An interesting study was carried out by Ratanaburee et al. [43] by selecting autochthonous LAB strains from fermented meat products with γ-amino butyric acid production (a compound associated with the regulation of diabetes, mental illness, and autonomic disorders). According to the authors, four selected strains (*P. pentosaceus* HN8, NH102, NH116, and *Lactobacillus namurensis* NH2) out of 14 isolates displayed potential to produce γ-amino butyric acid and be potentially used as probiotics. The authors of this study indicated that the four isolated probiotics were capable to resist the simulated gastrointestinal digestion assay.

It is worth mentioning that the influence of body temperature in the viability of potential probiotic strains was also tested. Barbosa et al. [29] indicated that non-significant effects were observed for the viability of *E. faecium* 85, 101, 119, and 120 counts for 120 min at 37 °C. In a similar way, Moreno et al. [30] showed that *L. lactis* subsp. *cremoris* CTC 204, *L. lactis* subsp. *hordiniae* CTC 483, *L. lactis* subsp. *cremoris* CTC 484, *L. plantarum* CTC 368, and *L. plantarum* CTC 469 can grow at 37 °C. Klingberg et al. [39] indicated that most of the isolates from Scandinavian-type fermented sausages displayed potential to grow at 37 °C after being freeze-dried.

Once the probiotics reach the gut, the capacity to adhere to enterocytes, auto-aggregation, as well as the high cell surface hydrophobicity are essential to improve the chances of successful colonization in small intestine. In this sense, many recent studies with isolates from meat products evaluated this crucial characteristic of probiotics. The study carried out by Wang et al. [28] was a valid example of this characterization. The strains *L. plantarum* CB9 and CB10 displayed higher capacity to adhere to the surface of SW480 cells than the *Weissella cibaria* CB12. A similar outcome was also obtained for hydrophobicity and auto-aggregation tests. Similarly, Topçu et al. [36] observed that the *P. pentosaceus* K7, K41, K44, K51, and K81 and *P. acidilactici* K99 displayed the higher values in the hydrophobicity, auto-aggregation, and cell adhesion assays from the microorganisms naturally found in pastırma. Regarding the isolates from Harbin dry sausages [27], *L. brevis* R4 was the isolate with the highest percentage in auto-aggregation, cell adhesion, and surface hydrophobicity assays. Another related study performed by Borah et al. [33] with *Staphylococcus* sp. DBOCP6 indicated that this bacterium displayed suitable levels of hydrophobicity and auto-aggregation. Contrastingly, in the experiment carried out by Dias et al. [35], the isolated LAB strains had intermediate cell hydrophobicity and auto-aggregation.

It is relevant to mention that these probiotic characteristics can vary among strains. This outcome was reported by Zommiti et al. [38]. These authors obtained the differences in the auto-aggregation and cell adhesion capacities of *E. faecium* strains isolated from Dried Ossban. While the MZF1 and MZF2 strains had the highest auto-aggregation capacity, the highest cell adhesion index was obtained from MZF5 strain. In a similar way, significant differences in the hydrophobic potential among isolated strains from fermented meat products were indicated by Ratanaburee et al. [43]. According to the authors, *P. pentosaceus* HN8 displayed the highest hydrophobic potential in comparison to other isolated strains *P. pentosaceus* NH102 and NH116 and *L. namurensis* NH2.

In another related study, the variation in the capacity to adhere to Caco-2 cells was obtained among 42 LAB isolates [26]. Most of the 42 isolates displayed weak or medium capacity to adhere to cell surface, except for *Enterococcus faecalis* 18156-3 and *L. casei* 12668-1. In the same line of thought, Klingberg et al. [39] observed that significant differences were observed among isolated microorganisms from Scandinavian-type fermented sausages. The strains *P. pentosaceus* DC12 and *L. salivarius* DC5 (isolated from Salami-type sausage and poultry salami, respectively) displayed the highest adhesion capacities while *L. farciminis* DC11 (isolated from a Salami-type sausage) displayed the lowest adhesion capacity. Another relevant example of the differences observed in the adhesion capacity among the isolated microorganisms from traditionally produced meat products was reported by Simonová et al. [42]. According to the authors, the strain with the highest adhesion index was *S. carnosus* SO2/F/2/5. Conversely, this study also indicated that the strains *S. xylosus* SO1/1M/2b and SO2/2M/2a showed minimal adhesion capacities. Additionally, the study carried out by Klayraung et al. [34] with three isolates of *L. fermentum* from fermented pork sausages indicated that the strain 3007 had the highest hydrophobicity index in comparison to the 2311 and 3010 strains.

In a recent study, the biofilm formation capacity of five LAB isolates was evaluated [30]. In this study, the authors observed that all strains formed biofilms and the highest capacity was reported for *L. lactis* subsp. *cremoris* CTC204. Moreover, this strain displayed the highest response to MgSO_4_ (a factor involved in the stimulation of microbial enzyme activity and growth) in the culture medium. The capacity to produce biofilm was also evaluated by Landeta et al. [40] in LAB isolated from Spanish dry-cured sausages. According to the authors, *L. sakei* strains (Al-109, Al-112, Al-113, and Al-115, for instance) displayed the capacity to produce biofilm as well as other isolated LAB such as *L. coryniformis* Al-127 and *L. paracasei* Al-120.

The capacity to inhibit the growth of pathogenic bacteria is another relevant probiotic activity. In this sense, the evaluation of antimicrobial potential as well as the co-aggregation capacity has been evaluated to select the probiotic strains from meat products. For instance, *E. faecium* 120 was the strain with the highest antimicrobial activity against the pathogens *Listeria monocytogenes* 7946 and 7947, *E. faecalis* ATCC 29212 and DSMZ 13590, *L. innocua* 2030c and NTCT 11286, and *Staphylococcus aureus* ATCC 29213 [29]. Additionally, the authors indicated that the most probable mechanism to explain this strong effect in comparison to other isolates was due to the production of a bacteriocin.

The evaluation of antimicrobial activity of autochthonous *E. faecium* isolates found in *Sokobanja* sausage (traditional Serbian sausage) revealed that most of the strains displayed an inhibitory effect against *Pseudomonas* spp., *Proteus* spp., and *Escherichia coli* [31]. Conversely, the effect in *Enterobacter* spp. and *L. monocitogenes* was strain-dependent wherein intense inhibitory effects were obtained from strain sk8-4 and sk8-5, for instance. A similar outcome was reported by Zommiti et al. [38] who observed that all isolated *E. faecium* strains displayed high antimicrobial activity against *Listeria innocua* HPB13 and *Enterococcus faecalis* ATCC 29212, especially strains MZF1 and MZF5. According to the authors, one of the possible explanations for this effect can be related to the production of bacteriocin (Enterocin A, B, and P). However, none of the isolates displayed potential to inhibit the growth of *S. aureus* ATCC 25923, *E. coli* DH5a, *P. aeruginosa* PAO1, and *S. typhimurium* ATCC 14028. The experiment carried out by Klingberg et al. [39] also indicated the strain-dependent effect in the antimicrobial activity of probiotics isolated from meat products. In this study, the authors observed that *L. plantarum* MF1291 displayed antimicrobial activity against *Bacillus cereus*, *Escherichia coli*, *Listeria monocytogenes*, *Salmonella typhimurium*, *Shigella flexneri*, and *Yersinia enterocolitica*; however, the same antimicrobial activity was not observed for *L. plantarum* DC13. Moreover, *L. pentosus* MF1300, *L. plantarum/pentosus* MF1290, and *L. salivarius* DC5 were also strains with antimicrobial activity against these pathogenic bacteria.

The *Pediococcus* spp. strains isolated by Yuksekdag et al. [37] also displayed different antimicrobial activity against the *L. monocytogenes*, *E. coli* O-157:H7, and *Micrococcus flavus*. Although all *Pediococcus* strains inhibit the growth of *L. monocytogenes*, the inhibition of *E. coli* O-157:H7 growth was observed with two strains: *Pediococcus* Z9P and Z10P. Additionally, only *Pediococcus* Z13P was capable to inhibit the growth of *M. flavus*. A similar outcome was reported by Simonová et al. [42] who studied the antimicrobial activity of bacteriocins produced by *S. xylosus* and *S.carnosus* strains from a Slovak traditional sausages. According to the authors, the inhibitory effect of bacteriocins produced by all strains was observed against *Enterococcus avium* EA5 and *Pseudomonas* sp. SO1/1M/1/4 but only the bacteriocins produced by *S. xylosus* SO3/1M/1/2 and *S. carnosus* SO2/F/2/5 inhibited the growth of *L. innocua* LMG13568.

Babić et al. [41] reported that *L. plantarum* 1 K, *L. delbrueckii* 2 K, *L. mesenteroides* 6K1, and *L. acidophilus* 7K2 inhibited the growth of *E. coli* 3014 but a strain-dependent effect was observed for *Staphylococcus* spp. isolates. In this case, the strains *S. warneri* 3K1, *S. xylosus* 4K1, and *S. lentus* 6K2 prevented the growth of *E. coli* 3014 whereas the strain *S. auricularis* 7K1 had a slight inhibitory effect. The selected *L. fermentum* strains obtained from fermented pork sausages displayed potential to inhibit the growth of *S. aureus* TISTR 029, *E. coli* TISTR 780, and *Salmonella typhi* DMST 5784 [34]. Likewise, the study performed by Borah et al. [33] indicated that *Staphylococcus* sp. DBOCP6 inhibited the growth of *E. coli* MTCC40.

In the study carried out by Vieira et al. [32], the antimicrobial activity of *P. acidilactici* CE51 was evaluated against *L. monocytogenes* ATCC 19015. Moreover, the authors indicated that this effect was attributed to a bacteriocin produced by *P. acidilactici* CE51 after neutralizing and heating (5 min at 95 °C) the supernatant of fermentation broth of this bacteria.

Another relevant aspect related to the expected antimicrobial activity of probiotics is the co-aggregation with pathogenic microorganisms with eventual elimination in the feces. This aspect was evaluated by Wang et al. [28] for *L. plantarum* CB9 and CB10 and *W. cibaria* CB12 with *S. aureus* ATCC 25923, *Salmonella enterica* ATCC 13076, *E. coli* ATCC 25922, and *Shigella dysenteriae* ATCC 13313. Different from that observed for auto-aggregation, the co-aggregation of isolates was strain-dependent for *S. aureus* (with *L. plantarum* CB9) and *S. enterica* (with *L. plantarum* CB9 and CB10). Additionally, a similar co-aggregation capacity of *E. coli* and *S. dysenteriae* was reported for the three isolated strains.

In a similar way, the *P. pentosaceus* strains isolated from pastırma displayed different capacities to co-aggregate with *E. coli* ATCC 25922 [36]. The highest values were reported for *P. pentosaceus K44* while *P. pentosaceus* K41 and *P. pentosaceus* K41 displayed lower co-aggregation capacities. Dias et al. [35] carried out a related experiment with 32 *Lactobacillus* spp. strains isolated from pork sausages and observed that most of these strains co-aggregated with *E. coli*, *S. typhi*, and *L. monocytogenes*. In the experiment carried out by Yuksekdag et al. [37], the co-aggregation of *P. pentosaceus* with *L. monocytogenes* ATCC 7644 was strain dependent. Although all strains displayed co-aggregated potential, the highest percentage value was obtained with Z13P strain.

Another interesting aspect related to antimicrobial activity of potential probiotics isolated from meat products is their capacity to produce exopolysaccharides that can inhibit the formation and also induce the disruption of biofilms formed by pathogenic bacteria. The effectiveness of these compounds in producing *Leuconostoc citreum* and *L. mesenteroides* was explored by Abid et al. [47]. According to the authors, the exopolysaccharides produced by both microorganisms were capable to inhibit the formation of biofilms from *S. aureus* ATCC 25923, *E. coli* 25922, and *E. faecalis* 25912. Moreover, all potential probiotic strains also disrupted the biofilms formed by these pathogenic bacteria but at different degrees: *E. coli* 25922 and *E. faecalis* 25912 were more resistant to the exopolysaccharides produced by *L. citreum* and *L. mesenteroides* whereas *S. aureus* ATCC 25923 was more susceptible to these compounds.

Another decisive characteristic to select autochthonous strains as probiotic is their safety when these microorganisms are introduced in the diet and do not cause an infection. In this sense, the antibiotic susceptibility of potential probiotics was evaluated by many studies with autochthonous microorganisms isolated from meat products (Table 1). The study performed by Federici et al. [26] evaluated the antibiotic resistance of 42 LAB isolates and revealed differences among species and strains. On the one hand, *L. plantarum* 9202-3 and *Lactobacillus ssp. sakei* 9202-6 were sensible to ampicillin, clindamycin, chloramphenicol, erythromycin, gentamycin, and tetracycline. On the other hand, the *P. pentosaceus* 12971-2 and 60211-2, *P. pentosaceus* 60211-2, *Lactobacillus paraplantarum* 35156-5 and *Lactobacillus johnsonii* 35156-2 were resistant to several of the tested antibiotics. It is important mentioning that few of these isolates displayed genes related to antibiotic resistance. A related experiment with *Lactobacillus* isolates displayed a similar outcome in terms of antibiotic resistance among strains [35]. Most of the isolated *Lactobacillus* strains were resistant to ampicillin, chloramphenicol, and gentamicin whereas almost all strains were susceptible to erythromycin. Similarly, the experiment performed by Ratanaburee et al. [43] also indicated that *P. pentosaceus* HN8, NH102, NH116, and *Lactobacillus namurensis* NH2 were susceptible to cefoperazone, cephalothin, chloramphenicol, erythromycin, and penicillin G. However, these authors also observed that the isolated strains were resistant to ceftazidime, gentamycin, kanamycin, norfloxacin, polymyxin B, streptomycin, and vancomycin.

In a related experiment, Moreno et al. [30] indicated that the resistance to antibiotics on strains isolated from Brazilian meat products was strain-dependent. The authors indicated that *L. lactis* CTC 204 was the most sensible to erythromycin, clindamycin, tetracycline, vancomycin, and amoxicillin. Likewise, the evaluation of antibiotic resistance of potential probiotics isolated from cured beef revealed that *L. plantarum* (CB9 and CB10) strains were sensible to ampicillin, tetracycline, chloramphenicol, erythromycin, roxithromycin, and lincomycin [28]. Conversely, *W. cibaria* CB12 displayed resistance to several antibiotics.

The experiment carried out by Landeta et al. [40] indicated that the resistance of LAB isolated from Spanish dry-cured sausages to antibiotics was species- and strain-dependent. Regarding the differences among species, several *E. faecium* were resistant to penicillin G and tetracycline whereas many *L. casei* were susceptible to these antibiotics. In the case of strain susceptibility, the *L. casei* Al-123 and Al-144 were resistant to tetracycline whereas *L. casei* Al-125, Al-134, and Al-139 were susceptible to this antibiotic. A similar outcome was reported for *L. fermentum* isolated from Fermented pork sausages [34]. While the strains 2311 and 3010 were resistant to ampicillin, gentamycin, and trimethoprim, the strain 3007 was susceptible to these antibiotics. It is also relevant to mention that these three strains were susceptible to erythromycin, kanamycin, quinipristin, rifampicin, streptomycin, and tetracycline.

Babić et al. [41] evaluated the antibiotic resistance of isolated bacteria from Slavonski kulen sausage and noticed that *L. acidophilus* 7K2, *L. delbrueckii* 2 K, *L. mesenteroides* 6K1, *L. plantarum* 1 K, *S. warneri* 3K1, and *S. xylosus* 4K1 were susceptible to erythromycin, gentamycin, and neomycin. Conversely, the strains *S. lentus* 6K2 and *S. auricularis* 7K1 were resistant to at least one of these antibiotics. The study performed by Yuksekdag et al. [37] also indicated differences in the susceptibility to antibiotics among *P. pentosaceus* strains. While the isolates Z9P, Z10P, and Z11P were susceptible to penicillin and ampicillin, the strains Z12P and Z13P were resistant to these antibiotics. It is also important to mention that all strains were susceptible to at least four antibiotics.

In the case of *E. faecium* strains isolated from Sokobanja sausage, all strains (except for sk8-1 and sk8-17) displayed low resistance to the amoxicillin, cefalexin, ceftriaxone, erythromycin, ofloxacin, penicillin, and tetracycline [31]. A related experiment carried out by Simonová et al. [42] indicated that all isolated LAB from Slovak traditional sausages were susceptible or had minimal resistance to amoxicillin, ampicillin, chloramphenicol, clindamycin, erythromycin, gentamycin, lincomycin, methicilin, neomycin, novobiocin, rifampicin, tetracycline, tobramycin, and vancomycin. Similarly, the *E. faecium* strains MZF1, MZF2, MZF3, MZF4, and MZF5 studied by Zommiti et al. [38] were susceptible to ampicillin, chloramphenicol, gentamicin, tetracycline, and vancomycin. Likewise, Borah et al. [33] indicated that *Staphylococcus* sp. DBOCP6 isolated from Indian fermented meat was susceptible to ampicillin, ciprofloxacin, clindamycin, erythromycin, gentamicin, kanamycin, tetracycline, and vancomycin. Additionally, the evaluation of antibiotic resistance of *P. acidilactici* CE51 to different antibiotics revealed that the isolate was susceptible to ceftazidime, clindamycin, erythromycin, oxacillin, penicillin G, and tetracycline but was resistant to ciprofloxacin, gentamicin, and vancomycin [32].

The safety of a probiotic also involves aspects such as the production of biogenic amines, the presence of virulence factors and hemolytic activity. In this regard, the Moreno et al. [30] evaluated the biosynthesis of biogenic amines potential of *Lactococcus* spp. and *Lactobacillus* spp. isolates and observed that *L. lactis* subsp. *cremoris* CTC 204 and *L. plantarum* CTC 368 displayed the lowest levels of cadaverine, histamine, putrescine, spermidine, and spermine among all isolated strains. All autochthonous *E. faecium* strains evaluated by Petrović et al. [31] did not produce histidine. However, the strains sk8-1 and sk8-17 produced tyrosine. Similarly, none of the 46 LAB strains isolated by Landeta et al. [40] produced histamine, putrescine, or cadaverine but all *E. faecium* strains were producers of tyrosine. Additionally, a related experiment with *S. xylosus* strains obtained from Slovak traditional sausages indicated these isolates did not produce cadaverine, histamine, phenylethylamine, putrescine, tryptamine, and tyramine [42]. Conversely, the production of phenylethylamine, tryptamine, and tyramine was reported in the strain *S. carnosus* SO2/F/2/5 from this study. The experiment carried out by Barbosa et al. [29] indicated that *E. faecium* strains 85, 101, 119, and 120 did not show amino acid decarboxylase activity. A similar lack of decarboxylase activity was reported by Dias et al. [35] for *Lactobacillus* spp. strains and by Zommiti et al. [38] for *E. faecium* strains.

Another relevant aspect related to the evaluation of safety is the presence of virulence factors. In this regard, the study performed by Moreno et al. [30] evaluated the thermonuclease, hemolytic, and gelatinase activities of *Lactococcus* spp. and *Lactobacillus* spp. isolates. According to authors, none of the selected strains had thermonuclease, hemolytic, and gelatinase activities. A similar outcome was reported by Wang et al. [28]. The *L. plantarum* (CB9 and CB10) and *W. cibaria* CB12 isolated from cured beef did not show hemolytic activity. Babić et al. [41] indicated that none of the isolated strains from Slavonski kulen sausage displayed enterotoxin activity.

In a related experiment with 46 LAB isolates, Landeta et al. [40] did not detect the presence of virulence factors among all LAB strains. Similarly, Dias et al. [35] observed that none of the *Lactobacillus* spp. strains isolated from pork sausages displayed hemolytic activity. The absence of hemolytic activity was also reported for *P. pentosaceus* and *L. namurensis* isolated from the fermented meat products studied by Ratanaburee et al. [43]. Likewise, the evaluation of hemolytic activity in *Staphylococcus* sp. DBOCP6 carried out by Borah et al. [33] did not indicate this isolated bacterium could be harmful. Conversely, the experiment carried out by Zommiti et al. [38] indicated that some *E. faecium* strains isolated from dried Ossban displayed virulence factors. According to the authors, the strains MZF2, MZF3, and MZF5 did not show virulence factors whereas virulence factors were detected in MZF1 and MZF4 strains.

Finally, it is also relevant to mention that thermotolerant probiotics can also be found in cooked meat products [44]. In this case, six *E. faecium* strains (UAM1, UAM2, UAM3, UAM4, UAM5, and UAM6) were isolated from Vienna sausages and only the UAM1 strain displayed probiotic potential.

The autochthonous LAB of meat products are the predominant group that better fits the requirement of probiotic selection criteria proposed by health authorities. Moreover, the presence of these microorganisms with high potential to be used as probiotics in the production of meat products strengthens the hypothesis that the autochthonous microbial population is a valuable source of probiotics for the production and development of functional meat products.

### 2.2. Identification Probiotic Strains

Along with the techniques used to characterize the probiotic activity of autochthonous strains from meat products, the identification at strain level is necessary to ensure the use of the exact microorganism [48]. For instance, Federici et al. [26] characterized the specific primers (D8635 and Coc) for the identification of 42 LAB isolates using the RAPD-PCR method. This protocol was also applied in the identification of *Lactobacillus* spp. and *Lactococcus* spp. isolated from different meat products [30], LAB and *Staphylococcus* spp. in Slavonski kulen sausage [41], and LAB in Scandinavian-type fermented sausages [39].

The use of 16S rDNA sequencing was also employed in the identification of *P. pentosaceus* R1, *L. brevis* R4, *L. curvatus* R5, and *L. fermentum* R6 isolated from Harbin dry sausages [27]. Likewise, this method was used by Wang et al. [28] for the identification of *L. plantarum* (CB9 and CB10) and *W. cibaria* CB12, by Petrović et al. [31] for *E. faecium* (sk6-1 and -17; sk7-5, 7 and 8; sk8-1, 2, 4, 5, 7, 12, 13, 17 and 20; sk9-3, 11 and 15; sk10-1, 7, 10 and 12) isolates, and by Vieira et al. [32] for *P. acidilactici* CE51. Klayraung et al. [34], Dias et al. [35], and Pennacchia et al. [46] applied the 16S rDNA sequencing technique to identify *Lactobacillus* spp. isolated from different meat products. Similarly, LAB and bifidobacteria strains were identified using this technique by Landeta et al. [40], Ratanaburee et al. [43], and Ruiz-Moyano et al. [45]. The experiments carried out by Topçu et al. [36] and by Yuksekdag and Aslim [37] identified *P. pentosaceus* and *P. acidilactici* and *P. dextrinicus* at strain level as well as Hernández-Alcántara et al. [44] and for *E. faecium* strains. In the case of *Staphylococcus* spp., Simonová et al. [42] identified the probiotic strains of *S. xylosus* and *S. carnosus*. In the same line, Borah et al. [33] used the 16S rDNA sequencing method to identify *Staphylococcus* sp. DBOCP6. It is worth mentioning that Zommiti et al. [38] performed the identification of *E. faecium* strains using a matrix-assisted laser desorption ionization-time-of-flight mass spectrometry (MALDI-TOF MS).

## 3. Application of Autochthonous Probiotics in Meat Products

The use of autochthonous probiotic bacteria as starter cultures in the production of fermented meat products also complied with the technological requirements: tolerance to stressors (reduced aw, for instance), the production of desired compounds (such as lactic acid, peptides and volatile compounds), inhibition of competitive microbiota (especially pathogenic bacteria), the preservation or enhancement of expected sensory attributes, and the low capacity to produce toxic compounds (such as enterotoxins and biogenic amines) are the most relevant aspects to define the viability of starter culture [25,49,50,51,52].

Some recent studies have provided a detailed view of the influence and the role of autochthonous probiotic strains in the processing of fermented products (Table 2). The use of autochthonous probiotic bacteria displays successful colonization of meat mass at the beginning of processing (fermentation stage), which prevails throughout the processing. The rapid growth, in the beginning, is a decisive aspect related to the production of meat products with autochthonous probiotics strains and prevents the growth of other microorganisms. For instance, Campaniello et al. [53] indicated that the counts of probiotic *L. plantarum* 178 increased in the beginning of the ripening period (from 7 to 8 log CFU/g) and remained stable until the end of processing in a Sweet Calabrian salami. High counts of LAB in meat products at the end of processing were reported by other authors using autochthonous probiotic starter cultures such as *L. plantarum* IIA-2C12 [54], *L. plantarum* IIA-2C12, and *Lactobacillus acidophilus* IIA-2B4 [55], *L. plantarum* L125 [56], *L. sakei* 8416, and *L. sakei* 4413 [57], *P. acidilactici* SP979 [58], and with a mix of ten strains of *L. plantarum* [35].

Another relevant aspect of the microorganisms that grow along with LAB with major technological relevance is the coagulase-negative staphylococci group. These microorganisms are directly involved in the modification of color by reducing nitrate intro nitrite that will eventually be converted into NO and form the nitrosomyoglobin pigment (characteristic cured color of fermented meat products) [59]. The growth of staphylococci group during the processing of a Sweet Calabrian salami, along with autochthonous probiotic LAB, was reported by Campaniello et al. [53]. At the end of processing of each fermented meat product, the LAB populations were in the range of 7–10 log CFU/g. Similarly, Pavli et al. [56] reported *Staphylococci* (4–5 log CFU/g) group as one of the main microorganisms during the processing of pork fermented sausage. Consequently, characteristic color of fermented meat products (especially redness) can be improved. Particularly for the improvement of redness, *L. plantarum* IIA-2C12 and *L. acidophilus* IIA-2B4 increased this quality indicator in comparison to control (without starter culture) in fermented beef sausages [55].

Although there is no current consensus about the ideal probiotic load in meat products to ensure health benefits, probiotics in meat products prevail during storage. The study performed by Pavli et al. [56] indicated that counts of probiotic strain (*L. plantarum* L125) were above 6 log CFU/g during 160 days of refrigerated storage either at 4 or 12 °C. This result is an important outcome to strengthen the role of autochthonous probiotic strains in the production of fermented meat products by indicating the survival of probiotic strains after long storage periods.

**Table 2:** Influence of probiotic strains as starter cultures in meat products.

Probiotic Microorganisms	Meat Product	Inoculum Count and Processing Conditions	Influence on Meat Product Quality Indicators	Ref.
*L. plantarum* 178	Sweet Calabrian salami	10 log CFU/g; stewing stage for 4 h at 22 °C and RH of 99%; drying stage for 7 h at 22 °C and RH of 65%; intermediate drying/ripening stage for 4 days from 20 to 15 °C, and RH from 67% to 73%; first ripening stage for 5 days at 15 °C and RH of 71%; second ripening stage for 5 days at 13 °C and RH of 73%, and final ripening/maturation stage for 15 days at 12 °C and RH of 75%	Increased LAB count; reduced pH; inhibited enterobacteria growth	[53]
*L. plantarum* IIA-2C12	Fermented lamb sausage	9 log CFU ^1^/mL; drying for 1 day 25 °C, cold smoking for 3 days at 27 °C	Reduced pH, aw ^2^ and *Escherichia coli* count; increased LAB ^3^ count, acidity, lactic acid content, and sensory acceptance	[54]
*L. plantarum* IIA-2C12 and *Lactobacillus acidophilus* IIA-2B4	Fermented beef sausage	9 log CFU/g; conditioning for 24 h at 27–29 °C and RH ^4^ 88–90%, cold smoking (three times) for 4 h (12 h in total) at 27–29 °C, and fermentation for 24 h at RT ^5^	Reduced pH, lipid oxidation, hardness, *Staphylococcus aureus* and *E. coli* counts; increased acidity, color, LAB count and volatile compounds; not meaningful changes on fatty acid profile, aw and sensory attributes	[55]
*L. plantarum* L125	Pork fermented sausage	8 log CFU/g; fermentation for 4 days; ripening for 8 days	High counts of LAB and staphylococci; increased redness, raw odor and acidic taste; reduced pH and aw; final product was microbiologically safe	[56]
*L. sakei* 8416 and *L. sakei* 4413	Beef and pork fermented sausage	7 log CFU/g; fermented for 6 days from 20 to 15 °C, RH from 95 to 80% and air velocity from 0.7 to 0.5 m/s, smoked for 3 h; ripened for 21 days at 15 °C, RH 80% and air velocity at 0.05–0.1 m/s	Increased LAB count; absence of *L. monocytogenes* and presumptive *E. coli* O157; reduced pH and aw	[57]
Mix with 10 *L. plantarum* strains	Fermented pork sausage	7 log CFU/g; 30 days at 10 °C	Increased LAB count; reduced *S. typhi and L. monocytogenes* counts, and pH	[35]
*P. acidilactici* SP979	Spanish *salchichón*	7.5 log CFU/g; 10 °C and 80% RH for 22 days at 12 °C and 70% RH for 26 days	Increased moisture and protein content; reduced pH, lipid content and oxidation,	[58]
*P. pentosaceus* HN8 and *L. namurensis* NH2	Thai fermented pork sausage (Nham)	6 log CFU/g for each strain; fermented for 4 days	Reduced biogenic amines and cholesterol contents	[60]

^1^ CFU: colony forming unit; ^2^ aw: water activity; ^3^ LAB: lactic acid bacteria; ^4^ RH: relative humidity; and ^5^ RT: room temperature.

A characteristic effect of LAB growth in the fermentation of meat products is the gradual pH drop during processing due to the production of lactic acid. This characteristic effect was reported in recent studies (Table 2) with meat fermentation by autochthonous probiotic LAB, such as reported by [54] in lamb sausage fermented with *L. plantarum* IIA-2C12. In this study, the inoculation of autochthonous probiotic bacteria increased the content of lactic acid in comparison to non-inoculated sausage (3.0% vs. 2.0%, respectively). In accordance with this scenario, the pH of meat products fermented with autochthonous probiotic was reduced in comparison to fresh meat mass prior to fermentation stage.

This drop of pH occurred with the fermentation of a pork meat sausage with *L. plantarum* L125 wherein a drop from 6 to below 4.5 was seen [56]. Moreover, non-significant difference was indicated in comparison to control (using commercial starter culture with *P. pentosaceus* and *Staphylococcus carnosus*). Likewise, the similar low pH (between 4.1 and 5.5) in the final product was also reported with other fermented meat products [53,55,57]. Conversely the experiment carried out by Ruiz-Moyano et al. [58] indicated that the addition of *P. acidilactici* SP979 did not significantly alter the pH of Spanish *salchichón* (final pH around 6.0). Likewise, Dias [35] obtained a final pH of 5.7 in fermented pork sausage inoculated with a mix of 10 *L. plantarum* strains after 30 days at 10 °C.

Another aspect related to the successful colonization of autochthonous probiotic strains during the fermentation of meat products is the inhibition of competitive microbiota (natural and contaminating). In this sense, recent studies indicated a similar or improved capacity to inhibit the growth of pathogenic microorganisms. The inhibitory effect was reported for *L. plantarum* IIA-2C12 and *L. acidophilus* IIA-2B4 that inhibited the growth of *E. coli*, *Salmonella* spp., and *S. aureus* in fermented beef sausage [55]. A similar antimicrobial effect against pathogenic groups of microorganisms was reported by Campaniello et al. [53] during the processing of Sweet Calabrian salami. The authors observed that Enterobacteria counts were reduced to non-detectable levels during the processing as well as for Clostridia, *E. coli*, *Salmonella* sp., and *L. monocytogenes*. The study performed by Dias et al. [35] indicated a reduction in the counts of *S. typhi* and *L. monocytogenes* in 30 days at 10 °C.

Likewise, *Brochothrix* spp., *Enterobacteriaceae*, *L. monocytogenes*, *Pseudomonas* spp., yeasts, and molds were below the detection limits in the pork sausages inoculated with *L. plantarum* L125 [56]. The experiment carried out by Pragalaki et al. [57] indicated the absence of *L. monocytogenes* and presumptive *E. coli* O157 in sausages elaborated with autochthonous probiotic strains *L. sakei* 8416 and *L. sakei* 4413 and in control (spontaneous fermentation) treatment. It is relevant mentioning that the outcomes reported in fermented meat products in relation to the antimicrobial activity are in accordance with the information indicated by the in vitro assays for the characterization of probiotic activity (Table 1).

Water activity (aw) is another important processing variable that influences the growth and metabolism of microorganisms in food, particularly when values below 0.9 are obtained during processing [61]. Although reaching this threshold is an important condition to inhibit the growth of spoilage and pathogenic microorganism and extend the shelf life of food, probiotic microorganisms are subjected to the same condition too. Differently than observed for other microorganisms, the selected autochthonous probiotic bacteria prevail in this condition and compose the majority of the microbial population in final products and during the storage period [56]. Other studies indicated a similar scenario where autochthonous probiotic strains (evaluated as LAB) were the main group of microorganisms: *L. sakei* 8416 and *L. sakei* 4413 at aw of 0.86 [55], *L. plantarum* 178 at aw < 0.85 [53], *L. sakei* 8416 and *L. sakei* 4413 at aw of 0.88 [57], *P. acidilactici* SP979 at aw of 0.90 [58], and with the combined use of 10 strains of *L. plantarum* at aw of 0.94 [35].

In terms of sensory evaluation, the use of autochthonous probiotic strains as starter cultures preserved or enhanced the sensory characteristics. For instance, Arief et al. [54] indicated that lamb sausage fermented with *L. plantarum* IIA-2C12 received higher score for aroma, color, and texture than the sausage elaborated without a starter culture. In a posterior study, the same group indicated similar acceptance of color, aroma, and texture among control (without) and two beef sausages inoculated with probiotics (*L. plantarum* IIA-2C12 and *L. acidophilus* IIA-2B4) [55]. Another relevant outcome indicated by this experiment was the influence of probiotic starter culture on the volatile compounds of the final product. According to the authors, the main influence was observed in the composition of volatile fraction, which suggested the influence in the metabolic process that generated the volatile compounds. For instance, the generation of acetic acid was enhanced in sausages elaborated with probiotics in comparison to control while an opposite effect was reported for ethyl alcohol.

In a study with the probiotic strain *L. plantarum* L125 in the production of pork sausage, a significant increase in the scores of redness, raw odor, and acidic taste during processing and storage in comparison to sausage produced without a starter culture was found [56]. The other sensory attributes (odor; taste; appearance; texture; paleness and oily appearance; smoking odor; acidic, aftertaste, juicy, salty, sweet, and spicy taste) were not affected by the probiotic starter culture. A similar outcome was reported for the use of *P. acidilactici* SP979 in Spanish *salchichón* where only the color was affected by the probiotic culture and no significant effect was reported for other attributes (flavor, taste, texture, odor, and acceptability) [58].

In addition to the effect in technological properties, the autochthonous probiotic starter cultures can also influence the cholesterol and biogenic amine content, as indicated by Kantachote et al. [60]. According to the authors, the mixed starter culture of *P. pentosaceus* HN8 and *L. namurensis* NH2 caused a reduction in seven biogenic amines (cadaverine, histamine, β-phenylethylamine, putrescine, spermidine, spermine, and tyramine) and also reduced the total cholesterol content in comparison to control and commercially produced *Nham* (a traditional Thai fermented pork sausage). A related experiment with *P. acidilactici* SP979 in the production of Spanish *salchichón* indicated no significant effect in the accumulation of biogenic amines after the ripening period [58]. In the context of food processing, autochthonous probiotic strains can be applied in the production of fermented meat products. Many advantages can be cited: fast and persistent colonization during and after processing, inhibition of competitive microorganisms (especially pathogenic bacteria in both in vitro tests and meat product), and preservation or enhancement of sensory properties.

## 4. Conclusions

The autochthonous microorganisms found in meat products have great potential to be applied as probiotic starter cultures. Consequently, meat products produced with starter cultures can be improved beyond their current use for a functional food market (especially for thermally treated meat products with thermotolerant strains) that has been growing in the last years. LAB plays an important role in this specific category of starter culture for the meat industry because of their probiotic potential (resistance to digestion, colonization of small intestine and safety aspects) and satisfactory characteristic from a meat processing point of view (fast colonization of meat mass, development of characteristic sensory attributes, as well as viability during storage).
